# Progress in Surgery

**Published:** 1898-10-22

**Authors:** 


### Progress in Surgery.

### SURGERY OF THE INTESTINES.

**Intestinal Anastomosis** is facilitated, according to Halsted,[1] by inserting into the divided ends of the intestine a sausage-shaped rubber bag, which can be inflated with air before the stitches are applied, and subsequently withdrawn before the stitches are tied. He claims the following advantages: (1) The vermicular action of the bowel is arrested over the bag, and the stitches can, consequently, be placed at regular and proper intervals. (2) The distended bag unrolls and spreads out to a fine edge, the inverted raw edge of the intestine, and enables the operator to place the stitches with great precision at the desired distance from the edge. (3) If distended intestine is to be sutured to collapsed intestine (in strangulated hernia, &c.), or intestine of larger to intestine of smaller bruren (jejunum to ileum, &c.), the smaller may easily be expanded to fit the larger piece. (4) Very little handling of the intestine itself by the operator is necessary. The tube from bag to syringe is used as a handle to rotate and elevate the parts to be united. (5) The cylinder takes the place of at least two assistants. The operation could readily be performed without an assistant. (6) It prevents escape of intestinal contents, and hence dispenses with the injurious clamp or the fingers of assistants. (7) The entire operation, exclusive of suture of the abdominal wall, can be performed on dogs in five or six minutes, and probably in less time.

Shelton Horsley[2] describes the following method of anastomosis: The ends of the divided bowels are placed side by side, opening in the same direction and being in contact along their free surfaces, opposite the attached mesentery. An artery forceps, inserted in the ends and clamped, holds them in this position. A finger of the left hand is inserted into one end of the intestine, and the thumb into the other, and over them, as a bobbin, a Cushing suture of silk in an ordinary cambric needle is begun. The first stitch approximates the portion of the two limbs of the intestine near the mesenteric attachment. The suture is then carried obliquely for about two inches to the border opposite the mesenteric attachment, and continued over the other side, where it stops at a place corresponding to its point of beginning. Here the needle is left on the thread, and an artery forceps seizes it where it emerges at the last stitch. This keeps the sutures tight. The bowel is now partly everted, exposing a U-shaped septum, grasped by the artery forceps first applied. The septum is cut away with curved scissors, leaving a margin of a third of an inch. An overhand suture of silk in a curved needle is then begun at one edge of the "shelf" left by cutting away the septum, and is carried through all the intestinal coats. When the suture reaches the end of this shelf, it is continued by slightly invaginating the rest of the resected ends, which consists of about one-fourth of the entire circumference. It terminates at its point of beginning. The first line of sutures is finished by continuing it about a quarter of an inch from the overhand suture. The incision in the mesentery is closed, and the operation is completed.

**Typhoid Perforation.**—Monod and Vanverts[3] have formed the following conclusions: (1) The results of surgical interference in general peritonitis from intestinal perforation in typhoid fever are not encouraging (2) The general results of operation are, however, better than expectant treatment, which has a mortality of 95 per cent. (3) The chances of success are greater when the perforation is produced at a late period in the typhoid fever, and especially during convalescence or at the end of a relapse. (4) Operation should be performed as soon as possible after the diagnosis of a perforation has been made. (5) The effects of surgical interference are excellent so far as occlusion of the perforation is concerned; much less satisfactory as regards the survival of the patient. (6) The causes of failure are numerous: progressive peritonitis, the production of fresh perforation, the bad general condition of the patient. (7) If the patient is in a very feeble state, or collapsed, it is better to abstain from operating. (8) Intervention generally means a median laparotomy and simple suture of the perforation. Intestinal resection with the formation of an artificial anus, is reserved for those cases in which the lesions are more complex. Free flushing and drainage terminate the operation. (9) In cases of encysted peritonitis, we should be content with making an incision into the purulent collection, and provide free drainage. We sometimes can find and suture the perforation. Finney[4] reports three cases with one recovery, and concludes that: (1) Of all the signs of perforating typhoid ulcer, most reliance is to be placed upon the development during the third or fourth week of severe, continued abdominal pain, with nausea and vomiting, and at the same time a marked increase in the number of white blood-corpuscles. (2) The surgical is the only rational treatment of perforating typhoid ulcer. (3) There is no contra-indication to the operation, except a moribund condition of the patient.

**Acute Intestinal Obstruction.**—McArdle[5] suggests the following points in treatment: Follow the engorged coil of intestines upward and downward, until the point of obstruction is reached, or turn out all the intestines. (2) Remove all fluid from Douglas's pouch and the loins by irrigation with sterile water. (3) Restore the colour of the bowels, and establish peristalsis by heating with neutral saline solution. The removal of the primary cause of intestinal obstruction is not always followed by relief of the symptoms. (4) Should there be difficulty in returning the intestines, elevate the pelvis in the Trendelenburg position, or, if necessary, open and wash out. (5) Above all, immediate operation is called for in all cases of complete obstruction of the bowel, with evidence of peritoneal effusion.

**Intussusception.**—Clubbe[6] records fifteen cases of intussusception, of which eight recovered and seven died. In three cases the intussusception was reduced by injection of oil without operation. Of the twelve cases subjected to laparotomy, five recovered. In one of the cases that recovered, four inches of the small intestine were resected, and this in a child under twelve months. The author has previously reported seven laparotomies with four recoveries. Taking the two series together, we get nineteen laparotomies with nine recoveries and ten deaths. He thinks that injections of warm oil should be given in all cases after the child is under an anæsthetic, even in cases of long standing where we know that it is impossible to complete the reduction by this means, for it always reduces the intussusception to a certain extent, and is the best and gentlest way; and in this way lessens the shock of the coming operation, because it lessens the manipulation of the intestines. It is especially useful in those cases in which we find the intussusception in the rectum; because if we do not use it we may find some difficulty in getting our fingers below the tumour to begin the squeezing process. Rutherford[7] records three cases unsuccessfully treated by laparotomy.

**Colotomy and Colostomy.** — Mosetig - Moorhof[8] describes a new modification in technique which he has found useful in certain cases. The disadvantage of the ordinary operation is that it does not prevent the entry of fæces into the distal part of the bowel, where they stagnate and tend to set up inflammatory troubles. If the distal portion is closed, the accumulated secretions may distend it and lead to ulceration. These difficulties may be partly surmounted by the formation of a spur, but this requires a long and freely movable colon and meso-colon. The author recommends his method when the ordinary means are inadvisable or impracticable. This consists in the usual operation of colostomy performed at one sitting, but preceded by partial occlusion of the distal portion of the bowel. A ligature is tied round this, occluding it to about one-half its diameter, and the bulging serous surfaces on either side are sewn together with interrupted stitches. An artificial constriction is thus produced which prevents the accumulation of fæces in the rectum. In attaching the gut to the body wall, the author first sews the serous and muscular coats of the intestine to the parietal peritoneum, and then passes the ordinary sutures through both bowel and abdominal wall. If, however, this would lead to considerable tension, he prefers to attach the intestine to the fascia of the external oblique, leaving the skin free, but shutting off the muscular planes from the risk of infection. Barling[9] records a case of excision of carcinoma of the sigmoid flexure, and Rutherford[10] a successful ease of resection of the cæcum for cylindrical epithelioma.

[1] Bulletin of The Johns Hopkins Hosp., Feb. 1898. [2] New York Med. Jour., Jan. 8, 1898, p. 66. [3] Med. Chron., Oct. 1897, p. 32. [4] Johns Hopkins Hosp. Bulletin, 1897, No. 74. [5] The Post Graduate, Mar. 1898, p. 225. [6] Brit. Med. Jour., Nov. 6, 1897, p. 1,335. [7] Glasgow Med. Jour., Feb. 1898, p. 119. [8] Epit. Brit. Med. Jour., Mar. 12, 1898; and Wein. Med. Presse, 1898, No. 3. [9] Birmingham Med. Rev., Mar., 1898, p. 129. [10] Glasgow Med. Jour., Feb. 1898, p. 117.

